# What are the determinants for individuals to undergo cardiovascular disease health checks? A cross sectional survey

**DOI:** 10.1371/journal.pone.0201931

**Published:** 2018-08-09

**Authors:** Ai Theng Cheong, Ee Ming Khoo, Su May Liew, Karuthan Chinna

**Affiliations:** 1 Department of Family Medicine, Faculty of Medicine and Health Sciences, Universiti Putra Malaysia, Serdang, Selangor, Malaysia; 2 Department of Primary Care Medicine, University of Malaya Primary Care Research Group (UMPCRG), Faculty of Medicine, University of Malaya, Kuala Lumpur, Malaysia; 3 School of Medicine, Faculty of Health and Medical Sciences, Taylor’s University Malaysia, Subang Jaya, Selangor, Malaysia; The University of Warwick, UNITED KINGDOM

## Abstract

**Background:**

There is a need to improve public’s participation in health checks for early identification of individuals at high risk of CVD for prevention. The objective of this study is to identify significant determinants associated with individuals’ intention to undergo CVD health checks. These determinants could be used to develop effective strategies to improve CVD health check participation.

**Methods:**

This was a cross sectional survey using mall intercept interviews. It was carried out in a hypermarket surrounded by housing estates with a population of varying socioeconomic backgrounds. Inclusion criteria were Malaysian nationality and age 30 years and older. The validated CVD health check questionnaire was used to assess participants’ intention and the determinants that influenced their intention to undergo CVD health checks.

**Results:**

A total of 413 participants were recruited. The median age of the participants was 45 years (IQR 17 years) and 60% of them were female. Participants indicated they were likely (45.0%) or very likely (38.7%) to undergo CVD health checks while 16.2% were not sure, unlikely or very unlikely to undergo health checks. Using ordinal regression analysis, perception of benefits, drawbacks of CVD health checks, perception of external barriers and readiness to handle outcomes following CVD health checks were the significant determinants of individuals’ intention to undergo CVD health checks.

**Conclusions:**

To improve individuals’ participation in CVD health checks, we need to develop strategies to address their perception of benefits and drawbacks of CVD health checks, the perceived external barriers and their readiness to handle outcomes following CVD health checks.

## Introduction

Cardiovascular disease (CVD) is a major health burden worldwide. It is the leading cause of death in the world and 80% of the CVD deaths are due to heart attacks and strokes [1]. More than three quarter of CVD deaths occur in low- and middle-income countries [1].

The majority of CVD are lifestyle-related, with modifiable risk factors accounting for 90% of the CVD risks [2]. Thus, the onset of CVD could be delayed or prevented and is amenable to early interventions such as lifestyle changes and pharmacological therapy [3–6]. Therefore, preventive care is important to reduce occurrence of CVD and its related health burden.

Health checks are part of the preventive strategy used in primary care to help identify patients at high risk of CVD for early intervention [7]. In countries especially the low- and middle-income countries where there is high prevalence of CVD and unawareness of cardiovascular risk factors [8], health checks are of paramount important for early detection and timely intervention.

Malaysia is a middle-income country. CVD has been the major cause of death since 1970s [9,10]. The prevalence of CVD risk factors is high and increasing [11–13]. However, more than half of the population with risk factors remain ignorant of their risk status [13]. Opportunistic health checks by health care providers are, therefore, a potentially useful means in detecting CVD risk factors in early stages. This will allow a prediction of their cardiovascular risk to be estimated for timely interventions. However, the uptake of health checks remains low in Malaysia, ranging from 20% to 40% [14,15].

Our earlier qualitative study had identified factors (perceived relevance of health checks, readiness to face screening outcomes, views of significant others and external barriers such as time, cost and accessibility) which people take into consideration during their decision-making to undergo health checks [16]. However, it did not provide information on the impact of these factors at population level [17]. A questionnaire survey is thus useful as it allows the determination of factors associated with the individuals’ intention to undergo CVD health checks [18]. In this cross-sectional study, we aimed to determine the significant factors influencing individuals’ intention to undergo health checks. It is hoped that this result could help guide the development of strategies promoting CVD prevention health checks at population level.

## Materials and methods

### Study design and setting

This was a cross sectional survey using mall intercept interviews [19]. This method was chosen as it allowed the researcher recruit community dwelling adults from varied socioeconomic backgrounds.

The study was carried out in a hypermarket located at an urban area, Cheras district of Kuala Lumpur, Malaysia. This area was surrounded by housing estates with private and public primary care clinics. This hypermarket was selected purposively for the wide range of population that attended the facility.

### Study population

All Malaysian aged 30 years and above who attended the hypermarket during the study period were invited to participate. The age group selected is accordance with the Malaysian Ministry of Health’s recommendation for screening of CVD risk factors [11]. Individuals with known history of stroke or coronary heart disease and those who could not understand the Malay language (Malaysia national language) were excluded from the study.

### Survey instrument

A questionnaire was developed in Malay language as this is a common language used by all ethnic groups in the country. (Refer S1 Appendix).

The questionnaire consisted of four sections: Section I was on participants’ socio-demographic information, history of CVD risk factors such as diabetes, hypertension, high cholesterol, overweight or obesity, smoking and family history of heart attack or stroke and their awareness of stroke and heart attack.

Section II was on participants’ previous experience of health checks.

Section III was on the determinants of CVD health check questionnaire [20]. This questionnaire was developed based on the findings from the earlier qualitative study and grounded to local and cultural context [16,20]. It was validated with good content and structural validity [20]. The item-content validity index ranged from 0.83 to 1.00. The factors loading of the items were more than 0.40 with good internal consistency (Cronbach’s alpha range: 0.66–0.85) [20]. The nine determinants measured are: Individuals’ belief that the course of CVD can be changed for the better, perceptions of self being at risk of CVD, perception of benefits of CVD health checks, perception of drawbacks of CVD health checks, preferred method for CVD prevention, individuals’ readiness to know the results of CVD health checks, individuals’ readiness to handle the outcomes following CVD health checks, external barriers and influence by significant others.

A Likert scale of 1 to 5 was used to indicate participant’s level of agreement with each item; score of 1 indicated “strongly disagree” and score 5 “strongly agree”.

Section IV was on participant’s intention to undergo CVD health checks. Participants were asked about their likeliness to undergo health checks in a specified timeline (within 3 months, 6 months or 1 year). A Likert scale (from score 1 “very unlikely” to 5 “very likely”) was used to denote the intention to undergo CVD health checks.

### Data collection

Recruitment of participants was carried out at the exit area of the hypermarket, where there was no health store, to minimize possible bias of recruiting shoppers who were likely to be aware of CVD risks if they were to visit a health store.

During data collection, a bunting was set up to advertise and attract shoppers. Participants were recruited in a convenient manner by two possible methods: The shoppers were either approached and invited by the researcher or they self-volunteered to participate.

Participants were briefed about the objective of the study and screened for eligibility. Those who agreed to participate were given the participants information sheet and informed consent were obtained. To minimize social desirability bias, participants were informed that there were no right, or wrong answers and a truthful answer would be the most appropriate.

Participants were encouraged to self-administer the questionnaire. In circumstances where participants had problems reading the questionnaire, for example those who had visual problems or had literacy issues, the researcher would provide assistance by reading aloud the questionnaire to the participants.

### Data analysis

All statistical procedures were performed using IBM SPSS Statistics software version 22.

In section III of the questionnaire, a Likert scale of score 1 to 5 was used to indicate participant’s level of agreement with each item. These were treated as continuous data and mean scores of each determinant was computed from its item’s score and used for analysis.

For the determinants of “individuals’ beliefs that the course of CVD can be changed for the better”, “perception of self being at risk of CVD” and “perception of benefits of CVD health checks”, a higher mean score denotes greater perception of the relevance of health checks. For the determinants of “perceived drawbacks of CVD health checks” and “preferred method for CVD prevention (healthy practice vs. medical measures)”, a higher mean score denotes lesser perception of the relevance of CVD health checks.

Readiness of participants to face the outcomes of CVD health checks included two determinants: individuals’ readiness to know the results of CVD health checks and individuals’ readiness to handle the outcomes following these checks. A higher mean score of these determinants denotes higher readiness in facing the outcomes of CVD health checks.

The external barriers towards CVD health checks were assessed in terms of time, cost, transportation and distance from the health check facilities. The higher the mean score of this determinant denotes a higher degree of barriers towards CVD health checks.

Influence from significant others included influence from doctors, family members, friends, employers and people around. The higher the mean score of this determinant denotes a higher degree of influence by others for the participants to undergo CVD health checks.

In section IV, participants were asked about their likeliness to undergo health checks in a specified timeline (within 3 months, 6 months or 1 year). Two outcome variables (dependent variables) were derived from this question: the degree of likeliness to undergo health checks and the likely timeline to undergo health checks. A higher intention of CVD health checks was reflected by a higher degree of likeliness to undergo CVD health checks. A higher intention of CVD health checks might also be reflected by the likely time they would attend the CVD health checks. The earlier time a participant decided to attend for health checks would indicate a higher degree of intention. These outcome data were ordinal data.

The degree of likeliness of a participant to undergo a health check was indicated by the highest score the individual answered, regardless of timeline. It was ranked from very unlikely (score of 1) to very likely (score of 5) (refer S1 Table). For ordinal regression analysis, the scores of 1, 2 or 3 were combined into one category (not sure, unlikely or very unlikely) due to the small numbers of these scores (refer S1 Table).

Similarly, the likely timeline for a participant to undergo health checks was indicated by the earliest time the participant would likely or very likely undergo a health check (score of 4 or 5 for the indicated time period) (refer S1 Table). For example, in section IV, if a participant chose a score of 3 within 3 months, a score of 4 within 6 months and a score of 5 within 1 year, the earliest time they would likely undergo a health check was within 6 months. With this, they would be classified into the category of likely to attend within 6 months. Four categories were derived: not sure or not likely to attend, likely to attend within one year, likely to attend within 6 months and likely to attend within 3 months.

Regression analysis was used to determine the relationship between the nine determinants and the two outcome variables (individuals’ degree of likeliness and the likely timeline to undergo health check). Two models (model 1 and 2), included all cases and had the two outcome variables analyzed using ordinal regression. Another two models (model 3 and 4), had 16 cases removed from the initial cases, to take into account of a possible Hawthorne effect. These 16 cases were participants who did not have any past health check experience but indicated an intention to undergo CVD health checks. Hawthorne effect meant the participant’s indication of an intention to undergo health checks might not be true, but a consequence of reactivity towards a socially desirable effect.

Two sets of regression analysis were performed, the first set without and the second set adjusted with the sociodemographic data (age, gender, ethnicity, working status, history of diabetes, high blood pressure, high cholesterol, overweight or obesity and past history of regular health checks).

The outcome variables were measured in an ordinal scale. Simultaneous ordinal regression was used in the analysis, which is useful in determining relative influence of each determinant on the outcome [21]. The complimentary log-log link function was used. Model fitness was assessed using the Pearson and Deviance goodness-of-fit measures [22]. The goodness-of-fit was acceptable. The test of Parallel lines was used to assess the proportional odds assumption. The assumption of test of Parallel lines was met for all the models (p-value >0.05) except model 4 with control of sociodemographic data (p = 0.013) (Refer S2 Table).

Multicollinearity of the determinant variables was examined using correlation coefficient of the determinant variables before regression analysis was performed. There were no multicollinearity detected and the correlation coefficient of the determinant variables was <0.85 [23] (Refer S3 Table).

### Ethical issues

The Medical Ethics Committee, University of Malaya Medical Centre (20145–274) approved this study.

## Results

A total of 877 shoppers were approached and 413 shoppers participated (response rate: 47.1%).

The median age of the participants was 45 years (IQR 17 years) and mean age was 50.5 years (SD 4.3 years). Table 1 shows details of the participants’ characteristics. More females (60%) participated. The majority of participants were Malays (53.3%) and Chinese (37.3%). Most had secondary education and above. Almost all participants reported awareness of heart attacks (98.3%) and strokes (99.0%). Half (53.5%) of the participants reported a history of having regular health checks at least once in two years. About 40% of the participants had a family history of CVD.

**Table 1:** Characteristics of participants in survey.

Characteristics		Frequency	Percentage
Gender, n = 413	Male	163	39.5
	Female	250	60.5
Age group (years), n = 413	30–39	136	32.9
	40–49	132	32.0
	50–59	80	19.4
	≥60	65	15.7
Ethnicity, n = 413	Malay	220	53.3
	Chinese	154	37.3
	Indian	24	5.8
	Others*	15	3.6
Education level, n = 413	Primary	16	3.9
	Secondary	186	45.0
	Tertiary	211	51.1
Marital status, n = 413	Never married	40	9.7
	Widow/widower	11	2.7
	Separated	16	3.9
	Married	346	83.8
Working status, n = 411	No	122	29.7
	Yes	289	70.3
History of co-morbidities, n = 412	Diabetes	40	9.7
	Hypertension	74	18.0
	Hypercholesteroleamia	71	17.2
	Overweight/obesity	87	21.1
	Smoking	38	9.2
Family history of CVD, n = 412	No	237	57.5
	Yes	175	42.5
Awareness of CVD, n = 412	Heart attack	405	98.3
	Stroke	408	99.0
Health check experience, n = 413	Having any form of health check experience	386	93.5
Regular health check experience, n = 411	At least once a year	158	38.4
	Once in two years	62	15.1

*indigenous

Most of the participants indicated that they were likely (45%) or very likely (38.7%) to undergo CVD health checks. 16.2% of the participants indicated that they were not sure, unlikely or very unlikely to undergo CVD health checks.

About 40.0% of the participants indicated that they were likely to attend the CVD health checks within 3 months. About one fifth of the participants indicated they were likely to attend the CVD health checks within 6 months and another one fifth within 1 year.

Table 2 showed the profile of the determinants. Generally, the participants agreed that the disease course can be changed for better outcomes and health checks were beneficial (mean score of 4.22 and 4.18 respectively). The mean score of perceived self at risk was just slightly above 3, which implied that on average the perception of CVD risk was not strong. The public preferred using healthy lifestyle such as healthy diet, exercise, tai chi, etc for CVD prevention than medical measures such as health check or medical treatment (mean score of 3.70). The participants were ready to know the results of CVD health checks and handle the outcomes following CVD health checks, in which the mean score of these two determinants was 4.10 and 3.94, respectively.

**Table 2:** The mean scores and 95% confidence intervals for degree of agreement for determinants.

Determinants examined	Mean score (95% CI)
Believe that the disease course can be changed for better outcomes	4.22 (4.17, 4.26)
Perceived self at risk of CVD	3.15 (3.08, 3.21)
Perceived benefits of health checks	4.18 (4.13, 4.23)
Perceived drawbacks of health checks	2.11 (2.05, 2.17)
Preferred method for CVD prevention (preferred healthy practice than medical measures)	3.70 (3.61, 3.78)
Readiness to know the results of CVD health checks	4.10 (4.05, 4.14)
Readiness to handle the outcomes following CVD health checks	3.94 (3.90, 4.00)
External barriers	2.31(2.26, 2.37)
Influence from significant others	3.85 (3.79, 3.90)

Generally, external barriers were not an issue for the participants, with the mean score being 2.31. However, about one fifth of the participants agreed or strongly agreed that cost was a barrier. Significant others played a role in influencing the public to undergo CVD health checks (mean score 3.85). A high proportion of participants reported that they would undergo CVD health checks following advice from doctors (93.4%), family members (82.5%), friends (70.2%) or employers (87.6%).

### Factors associated with the intention for CVD health checks

Table 3 shows the determinants of individuals’ intention for CVD health checks without adjusted for the sociodemographic data. In the analysis of all 413 participants, people’s perception of the benefits of CVD health checks, and external barriers were the significant determinants of both individuals’ degree of likeliness to undergo CVD health checks (model 1) and individuals’ likely timeline to undergo CVD health checks (model 2). Perceptions regarding drawbacks of these checks was also a significant determinant of model 1.

**Table 3:** Determinants of individuals’ intention to undergo health checks without adjusted for sociodemographic data.

	Analysis with all participants (n = 413)						Analysis with 16 cases removed for Hawthorne effect (n = 397)					
Determinants	Model 1: Degree of likeliness to undergo CVD health checks			Model 2: Likely timeline to undergo CVD health checks			Model 3: Degree of likeliness to undergo CVD health checks			Model 4: Likely timeline to undergo CVD health checks		
	β	SE	p	β	SE	p	β	SE	p	β	SE	p
	(95%CI)			(95%CI)			95%CI			(95%CI)		
Believe that the disease course can be changed for better outcomes	-0.035	0.156	0.824	0.076	0.152	0.616	-0.025	0.162	0.878	0.073	0.158	0.641
	(-0.340 to 0.271)			(-0.222 to 0.375)			(-0.342 to 0.292)			(-0.235 to 0.382)		
Perceived self at risk of CVD	-0.004	0.106	0.967	0.177	0.102	0.083	-0.022	0.109	0.843	0.181	0.105	0.084
	(-0.212 to 0.204)			(-0.023 to 0.377)			(-0.236 to 0.193)			(-0.024 to 0.387)		
Preferred method for CVD prevention	0.029	0.087	0.742	-0.119	0.086	0.166	-0.010	0.090	0.914	-0.130	0.089	0.143
	(-0.142 to 0.199)			(-0.288 to 0.049)			(-0.187 to 0.167)			(-0.304 to 0.044)		
Perceived benefits of health checks	**0.526**	**0.183**	**0.004**	**0.442**	**0.177**	**0.012**	**0.547**	**0.189**	**0.004**	**0.447**	**0.182**	**0.014**
	**(0.168 to 0.884)**			**(0.096 to 0.788)**			**(0.176 to 0.917)**			**(0.090 to 0.804)**		
Perceived drawbacks of health checks	**-0.265**	**0.131**	**0.042**	-0.147	0.128	0.252	**-0.276**	**0.134**	**0.039**	-0.155	0.131	0.239
	**(-0.521 to -0.009)**			(-0.399 to 0.105)			**(-0.538 to -0.014)**			(-0.412 to 0.103)		
Readiness to know the result of health checks	0.222	0.187	0.234	-0.011	0.179	0.949	0.202	0.190	0.289	-0.038	0.182	0.834
	(-0.143 to 0.588)			(-0.362 to 0.339)			(-0.171 to 0.574)			(-0.394 to 0.318)		
Readiness to handle the outcomes following health checks	0.346	0.180	0.055	0.267	0.172	0.120	**0.552**	**0.189**	**0.004**	**0.386**	**0.180**	**0.032**
	(-0.007 to 0.699)			(-0.070 to 0.605)			**(0.181 to 0.923)**			**(0.033 to 0.738)**		
External barriers	**-0.489**	**0.151**	**0.001**	**-0.435**	**0.145**	**0.003**	**-0.469**	**0.161**	**0.004**	**-0.467**	**0.154**	**0.002**
	**(-0.785 to -0.193)**			**(-0.718 to -0.151)**			**(-0.784 to -0.154)**			**(-0.768 to -0.165)**		
Influence by significant others	0.086	0.135	0.524	0.238	0.128	0.063	0.113	0.139	0.415	0.253	0.131	0.053
	(-0.178 to 0.350)			(-0.012 to 0.489)			(-0.159 to 0.385)			(0.004 to 0.510)		

β: Estimates of regression coefficient; SE: Standard error; CI: Confidence interval

After removing the 16 cases for possible Hawthorne effect, four significant determinants were identified (model 3 and 4). The three determinants: individuals’ perception of the benefits of CVD health checks, individuals’ readiness to handle outcomes following health checks and external barriers were significant determinants of both individuals’ degree of likeliness to undergo CVD health checks (model 3) and individuals’ likely timeline to undergo CVD health checks (model 4). Perceptions regarding drawbacks of these checks remained a significant determinant of individuals’ degree of likeliness to undergo CVD health checks (model 3).

There were two possible relationships based on the positive or negative value of the β of the determinants. A positive value of the β indicates a positive relationship and a negative value indicates a negative relationship. Individuals’ perception of the benefits of CVD health checks had a positive relationship with their degree of likeliness and likely timeline to undergo CVD health checks. A higher degree of perceived health check benefits and readiness to handle outcomes were significantly associated with a higher degree of likeliness of the individuals to undergo CVD health checks and to undergo these checks within a shorter timeline.

The perceptions regarding drawbacks of these checks and external barriers had a negative relationship with the individuals’ degree of likeliness to undergo CVD health checks. The lower the degree of perceived health check drawbacks and external barriers was significantly associated with a higher degree of likeliness of the individuals to undergo CVD health checks and a higher likelihood of the individuals to undergo CVD health checks within a shorter timeline.

The 4 models seemed to explain 22.7%, 16.3%, 24.8% and 17.8% (corresponding to the pseudo-R^2^ of 0.227, 0.163, 0.248 and 0.178, respectively) of the observed variance in the individuals’ intention to undergo CVD health checks (Refer S2 Table). According to Cohen et al., the R^2^ value of 0.13 and 0.26 are proposed as medium and large effect sizes for the population [24]. The models in this study were useful in explaining the individuals’ intention to undergo CVD health checks.

Table 4 shows the determinants of individuals’ intention for CVD health checks adjusted for the sociodemographic data. After controlling for sociodemographic characteristic (age, gender, ethnicity, working status, history of diabetes, high blood pressure, high cholesterol, overweight or obesity and past history of regular health checks) and removing possible Hawthorn effect, individuals’ perceptions regarding benefits and drawbacks of health checks, individuals’ readiness to handle outcomes following health checks and external barriers were significant determinants of individuals’ degree of likeliness to undergo CVD health checks (model 3). For individuals’ likely timeline to undergo CVD health checks (model 4), the significant determinants were individuals’ readiness to handle the outcomes following health checks and external barriers. These two models (model 3 & model 4) explained 45.3% and 30.1% of individuals’ degree of likeliness to undergo CVD health checks and the individuals’ likely timeline to undergo CVD health checks respectively (Refer S2 Table).

**Table 4:** Determinants of individuals’ intention to undergo health checks adjusted for sociodemographic data^.

	Analysis with all participants (n = 412^#^)						Analysis with 16 cases removed for Hawthorne effect (n = 396^#^)					
Determinants	Model 1: Degree of likeliness to undergo CVD health checks			Model 2: Likely timeline to undergo CVD health checks			Model 3: Degree of likeliness to undergo CVD health checks			Model 4: Likely timeline to undergo CVD health checks *		
	β	SE	p	β	SE	p	β	SE	p	β	SE	p
	(95%CI)			(95%CI)			95%CI			(95%CI)		
Believe that the disease course can be changed for better outcomes	-0.105	0.166	0.527	0.035	0.154	0.820	-0.115	0.172	0.503	0.016	0.160	0.919
	(-0.429 to 0.220)			(-0.267 to 0.337)			(-0.453 to 0.223)			(-0.298 to 0.331)		
Perceived self at risk of CVD	-0.119	0.116	0.303	0.098	0.107	0.356	-0.116	0.119	0.330	0.117	0.109	0.286
	(-0.346 to 0.107)			(-0.110 to 0.307)			(-0.350 to 0.117)			(-0.097 to 0.331)		
Preferred method for CVD prevention	0.032	0.093	0.728	-0.068	0.089	0.444	-0.004	0.097	0.970	-0.087	0.092	0.343
	(-0.150 to 0.214)			(-0.242 to 0.106)			(-0.193 to 0.186)			(-0.267 to 0.093)		
Perceived benefits of health checks	**0.390**	**0.188**	**0.038**	0.302	0.176	0.086	**0.450**	**0.195**	**0.021**	0.309	0.182	0.089
	**(0.022 to 0.758)**			(-0.043 to 0.647)			**(0.069 to 0.832)**			(-0.047 to 0.666)		
Perceived drawbacks of health checks	**-0.471**	**0.141**	**0.001**	-0.267	0.137	0.050	**-0.497**	**0.145**	**0.001**	-0.280	0.142	0.048
	**(-0.747 to -0.195)**			(-0.535 to 0.001)			**(-0.782 to -0.212)**			(-0.558 to -0.002)		
Readiness to know the result of health checks	0.224	0.199	0.260	0.037	0.182	0.837	0.143	0.203	0.482	-0.016	0.187	0.933
	(-0.166 to 0.615)			(-0.320 to 0.395)			(-0.255 to 0.540)			(-0.381 to 0.350)		
Readiness to handle the outcomes following health checks	0.358	0.190	0.059	0.280	0.176	0.112	**0.513**	**0.202**	**0.011**	**0.463**	**0.187**	**0.013**
	(-0.014 to 0.730)			(-0.066 to 0.626)			**(0.117 to 0.908)**			**(0.098 to 0.829)**		
External barriers	-0.297	0.159	0.062	**-0.317**	**0.148**	**0.032**	**-0.335**	**0.170**	**0.048**	**-0.318**	**0.157**	**0.043**
	(-0.610 to 0.015)			**(-0.606 to -0.028)**			**(-0.668 to -0.003)**			**(-0.626 to -0.011)**		
Influence by significant others	0.155	0.140	0.268	0.211	0.130	0.105	0.207	0.144	0.150	0.249	0.134	0.064
	(-0.119 to 0.430)			(-0.044 to 0.467)			(-0.075 to 0.489)			(-0.014 to 0.511)		

β: Estimates of regression coefficient; SE: Standard error; CI: Confidence interval

^control for age, gender, ethnicity, working status, history of diabetes, high blood pressure, high cholesterol, overweight or obesity and past history of regular health checks

*Test of parallel lines p = 0.013

# one participant had missing data of history of history of diabetes, high blood pressure, high cholesterol, overweight or obesity

## Discussion

This cross-sectional survey was set up to identify determinants of individuals’ intention to undergo CVD health checks. The proposed models were useful in explaining individuals’ intention to undergo CVD health checks. Significant determinants identified were perception of benefits and drawbacks of CVD health checks, external barriers and readiness to handle outcomes following CVD health checks.

### Interpretation of findings and comparison to previous findings

A high proportion of participants indicated positive intention to undergo CVD health checks. This finding is consistent with another study, in which most people showed a positive intention towards CVD risk factor screening [25]. In our study, 45% of participants reported they were “likely” and 39% reported “very likely” to undergo CVD health checks. These “likely” and “very likely” responses could correspond to a moderate and high degree of intention, respectively. Locally, the National Health Morbidity Survey in 2011 reported about 38% of respondents had undergone health checks such as screening of blood pressure and blood sugar over the past 12 months [14]; this figure was similar to the high intention group in the present study. This suggests that people with high intention to undergo CVD health checks might realise their intention into action. In contrast, people with moderate intention might not realise their intention into action. This was supported by literature that showed attenders for screening were more likely to have definite intention than non-attenders [26].

A sensitivity analysis was conducted to test possible Hawthorne effect by removing 16 cases of participants who did not have any past health check experience but indicated intention to undergo CVD health checks. This analysis had resulted in an additional significant determinant (readiness to handle outcomes following health checks) to the initial analysis. As it is likely that the initial models were influenced by the Hawthorne effect, the added factor is likely to be a true determinant.

We found significant determinants for intention to undergo CVD health checks were positive perception of benefits and negative perception of drawbacks of CVD health checks, negative perception of external barriers and readiness to handle outcomes following CVD health checks. These findings were in line with some of the results found in previous research. The findings of positive perception of benefits of health checks were reported as significant predictors for intention to health checks in surveys based on the health belief model [27,28], and in a Dutch Health Care Consumer Panel survey, its result showed that the belief in the check that could contribute to a higher chance for aging healthily, was significantly associated with the willingness to participate in health checks [29]. Unpleasant screening procedures such as pain from finger stick tests have been reported as a barrier for health check participation [30], which was reflected by the perceived drawbacks of health checks in this study. It is important to emphasize the benefits of health checks and address the drawbacks of these checks when disseminating health check educational material to the public.

External barriers such as time constraints have been reported as significant factors that deterred public from attending health checks [27,29]. However, in the present study, participants were more concerned about cost rather than time, with only 1% of them indicating time was a barrier, compared to 20% for cost. Cost was raised as a concern in the utilization of private practice health care services in the Malaysia National Health Morbidity Survey [31]. A systematic review found that providing financial incentives for screening was an effective intervention to increase uptake of CVD health checks [32]. This could be a potentially useful measure to improve CVD health checks locally.

Previous research had shown that fear of health check results, and perception of health checks as being unnecessary worries, had reduced people’s intention to health check participation [26–29]. These factors are conceptually similar to readiness to know the result in the present study, where concerns could reflect a lack of readiness. We found individuals’ readiness to know the results was not a determinant of intention to undergo health checks, but the individuals’ readiness to handle the outcomes such as preparedness to take medication, preparedness to adjust lifestyle and preparedness to bear the cost of subsequent treatment following abnormal health checks was a significant determinant. Readiness to handle outcomes provides further understanding about the factors that people consider during the decision-making process, which has not been highlighted in the medical literature. Our findings show that there is a need to address individuals’ readiness to handle outcomes when developing interventions to improve CVD health checks.

There were inconsistent findings on the association of individuals’ perception of susceptibility and seriousness of disease with the intention or participation in health checks. We found the perception of self being at risk of CVD was not a significant determinant for intention to undergo CVD health checks. The mean score of this concept was 3.15, which suggested the perceived susceptibility of the disease was not high. However more than two-thirds of participants indicated they have moderate or high intention to undergo CVD health checks. It was uncertain whether this was a consequence of bias due to participants volunteering to take part in the study or differing cultural context. Future studies would be needed to verify this using probability sampling. Some studies reported health check attenders had a higher level of perceived susceptibility to disease [33], but many did not find this to be so [27,28,30,34,35]. Intervention studies that sent health risk appraisal questionnaire to participants showed that most people at risk who received the questionnaire did not turn up for CVD risk factor screening [36,37]. On the other hand, a Cochrane systematic review reported that there was a small effect with low quality evidence that suggested personalized risk communication increases uptake of screening tests, which was mainly for mammography and colorectal cancer screening [38].

### Recommendations/Implications

To improve uptake of CVD health checks, the four significant determinants (i.e. perception of benefits and drawbacks of CVD health checks, perception of external barriers and readiness to handle outcomes following CVD health checks) are important issues to address when developing strategies. For effective health communication, health materials should contain relevant information for the targeted group [39]. Hence health messages to the public must emphasize benefits of CVD health checks and address the drawbacks of these checks. Information that are important to the public such as side effects of medication, health check procedures or lifestyle management need to be included to reduce misconceptions and enhance readiness to face outcomes of health checks.

A systematic review had identified effective interventions to decrease external barriers such as financial incentives to individuals [32]. In this study, external barriers were shown to affect participation in health checks. There has been efforts to improve the uptake of CVD screening such as the provision of free vouchers to attend health screening by the Social Security Organisation (SOCSO) to its active members aged 40 years and above, and community outreach health check programmes [40,41] that help address some external barriers such as cost and accessibility. Members of the public who do not have access to these supports should be made aware regarding the availability of CVD health checks in government clinics, which is provided free of charge. Flexible government clinic appointment times, such as having the checks after office hours or at weekends, would probably increase public participation. However, there remains challenges for implementation such as availability of resources and healthcare personnel.

### Strength and limitations

This study was conducted in the community and it allowed the researcher to recruit the public from various backgrounds. However, due to the convenience sampling of survey population, there is a lack of representativeness of the study population to the general population. Future studies could use multi-stage sampling or random sampling for representative population and cover a wider population, including those in rural areas.

This study only included those who could speak or read Malay; this would probably exclude a number of people especially those who were older or of a poorer sociodemographic class. However, there was only one Indian participant who was excluded due to language issue. Therefore, the results of this study may be applicable to populations with similar sociodemographic characteristics.

## Conclusions

The significant determinants influencing people’s intention to undergo CVD health checks were the perception of benefits and drawbacks of CVD health checks, perception of external barriers and readiness to handle the outcomes following CVD health checks. It is important to address these determinants when developing interventions to increase CVD health checks to facilitate CVD screening uptake and ultimately offer opportunities to reduce CVD incidence.

## Supporting information

S1 Appendix The “determinants of intention to undergo CVD health checks” questionnaire. (PDF)

S1 Table Classification of the degree of likeliness and likely timeline to undergo health checks. (PDF)

S2 Table Summary results of pseudo-R^2^ and test of parallel lines for four models. (PDF)

S3 Table Correlation matrix for determinant variables in the model. (PDF)
