# Supplementary material for: What are the determinants for individuals to undergo cardiovascular disease health checks? A cross sectional survey
Source: PLoS One. 2018 Aug 9;13(8):e0201931. doi: 10.1371/journal.pone.0201931 (PMC6085058; doi:10.1371/journal.pone.0201931)
Supplement: S1 Appendix — (PDF) [file pone.0201931.s001.pdf]

S1 Appendix

Questionnaire

Kod No:............................
(kegunaan pejabat)
Office use

Sila jawab semua soalan dan isikan maklumat di bawah. Sila tanda (√) pada petak yang yang berkenaan.
Please fill in the information and answer all the questions below. Please tick (√) in the appropriate box.

Bahagian I :Maklumat peribadi
Demographic data

1. Umur : .... tahun
   Age *years old*

2. Tahun kelahiran : 19................
   Year of birth

3. Jantina: Lelaki ☐ Perempuan ☐
   Gender *Male* *Female*

4. Bangsa : Melayu ☐ Cina ☐ India ☐
   Ethnicity *Malay* *Chinese* *Indian*
   Lain-lain ☐ Sila nayatakan
   Others *Please specify* ……………………………………

5. Tahap pendidikan tertinggi anda:
   Your highiest level of educational
   - Pendidikan Tertiari (kolej, universiti) *Tertiary(college, university)* ☐
   - Menengah *Secondary* ☐
   - Rendah *Primary* ☐
   - Tiada pendidikan formal *No formal education* ☐

6. Status perkahwinan:
   Marital status
   - Berkahwin *Married* ☐
   - Bercerai atau berpisah *Divorced or separated* ☐
   - Balu *Widow/widower* ☐
   - Tidak pernah berkahwin *Never married* ☐

7. Adakah anda bekerja?
Are you working?

Ya (sekiranya ya, sila jawap soalan 8) ☐
Yes1 (If yes, go to question no.8.)

Tidak ☐
No

Sekiranya tidak, adakah anda
If no, are you

Menganggur ☐
Unemployed

Suri rumah tangga ☐
Housewife

Pesara ☐
pensioner

8. A) Sila nyatakan status majikan anda.
Please state your employer status.

Swasta ☐
Private

Kerajaan/separa kerajaan ☐
Government/semi-government

Kerja sendiri ☐
Self employed

B)Apakah pekerjaan anda?
What is your occupation?

Profesional *(doktor, jurutera, arkitek, ahli sains,ahli farmasi, guru, pentadbir/pengurusan, pekerja sosial, pengarah dll)1* ☐
***Professional** (doctor, engineer, scientist, pharmacist, teacher, manager, social worker, director etc.)*

Pekerja mahir dan perkeranian *(Artisan, kerani, mekanik, penyelia, peniaga dll)2* ☐
***Skilled worker and clerical workers** (artist, clerk, mechanic, supervisor, businessman etc)*

Pekerja separa mahir *(Petani, pekerja sektor pertanian, pekerja kilang dll)3* ☐
***Semi-skilled workers** (farmer, plantation worker, factory worker etc.)*

Pekerja tidak mahir*(buruh, pekerja am, pembantu rumah, pemendu teksi/bas/lori, pekerja kasual dll)4* ☐
***Non skilled worker** (labourer, maid, taxi/bus/lorry driver, casual worker etc.)*

9. Adakah anda menghidapi penyakit atau masalah kesihatan berikut?
Do you have the following diseases/conditions?

	Ya *yes*	Tidak *no*
Kencing manis *Diabetes*		
Darah tinggi *High blood pressure*		
Kolesterol tinggi *High cholesterol*		
Berat badan berlebihan/kegemukan *Overweight/Obesity*		
Merokok *Smoking*		

10. Pernahkah anda dengar mengenai penyakit serangan jantung sebelum ini?
Have you heard about heart attack?

Ya *yes* ☐ Tidak *no* ☐

11. Pernahkah anda dengar mengenai strok (angin ahmar) sebelum ini?
Have you heard about stroke?

Ya *yes* ☐ Tidak *no* ☐

12. Adakah sesiapa di antara keluarga anda (ibubapa atau adik-beradik) menghidapi sakit jantung/strok?
Is any of your immediate family members (parents or siblings) had heart attack /stroke?

Ya *yes* ☐ Tidak *no* ☐

Bahagian II Pengalaman tentang pemeriksaan kesihatan
Part II Experience of health checks

1. Pernahkah anda menbuat pemeriksaan kesihatan?
Have you undergone a health checks before?

Ya *yes* ☐ Tidak *no* ☐

Jika jawapan soalan 1 adalah YA, sila jawap soalan 2 ke 6. Jika jawapan adalah TIDAK, sila terus ke Bahagian III.
If the answer to question 1 is YES, please answer questions 2 to 6. If the answer is NO, please proceed to Part III.

2. Jika ya, bilakah pemeriksaan kesihatan terakhir dijalankan?
   If yes, when was your latest health check?

   Tahun: ………………. (contoh: 2011)
   year

   Bulan : …………….. .(contoh: Januari)
   month:

3. Di manakah anda menjalani pemeriksaan kesihatan terakhir ini?
   Where did you perform the health check ?

Klinik kerajaan<br>*Government health clinic*	☐
Klinik swasta<br>*Private clinic*	☐
Makmal perubatan (contohnya Pathlab, BP lab, *etc)*<br>*Clinical lab (e.g. Pathlab, BP lab, etc)*	☐
Kedai farmasi<br>*Pharmacy*	☐
Lain-lain<br>*Others* ………………………………………….	

4. Merujuk kepada soalan di atas, apakah penilaian kesihatan yang telah anda jalani? (Jawapan boleh lebih daripada satu)
What were the assessments performed in your latest health check? (You may tick more than one box)

Ditanya sejarah merokok<br>*Assessment of smoking history*	☐
Ditanya sejarah sakit jantung atau strok dalam keluarga<br>*Assessment of family history of cardiovascular diseases (stroke/heart disease/heart attack)*	☐
Ukuran tekanan darah<br>*Blood pressure measurement*	☐
Ukuran berat badan<br>*Weight measurement*	☐
Ukuran lilit pinggang<br>*Waist circumference measurement*	☐
Ujian darah kolesterol<br>*Blood test for cholesterol*	☐
Ujian darah gula<br>*Blood test for sugar*	☐

Lain-lain (sila nyatakan): ……………………………………………………….
Others (please specify) ……………………………………………………………………….

5. Mengapa anda melakukan pemeriksaan kesihatan tersebut?
Why did you perform the above health check?

Untuk mengetahui kesihatan diri<br>*To know my health status*	☐
Memenuhi syarat untuk bekerja<br>*Requirement for job*	☐
Memenuhi syarat untuk belajar<br>*Requirement for study*	☐
Rawatan susulan<br>*Follow up for treatment*	☐

Lain-lain (sila nyatakan sebab): …………………………………………………..
Others (please specify)

6. Berapa kerapkah anda menjalani pemeriksaan kesihatan?
How often do you perform health checks?

Kurang-kurangnya setahun sekali<br>*At least once a year*	☐
Sekali dalam 2 tahun<br>*Once in 2 years*	☐
Lebih daripada 2 tahun<br>*More than 2 years*	☐

Lain-lain
Others ……………………………………..

Bahagian III Persepsi terhadap penyakit CVD (contoh: penyakit jantung/strok) dan pemeriksaan kesihatan CVD.

- Soalan-soalan di bawah bertujuan untuk mendapat persepsi anda terhadap penyakit kardiovaskular dan pemeriksaan kesihatan untuk pencegahan penyakit kardiovaskular.

  -**Penyakit kardiovakular (penyakit CVD)** dalam soalan-soalan berikut adalah merujuk kepada **penyakit serangan jantung dan angin ahmar (strok).**

  -**Pemeriksaan kesihatan CVD** dalam soalan-soalan berikut **adalah pemeriksaan untuk mengesan risiko penyakit CVD seperti darah tinggi, kencing manis, masalah kolesterol tinggi, kegemukan, merokok dan kekurangan aktiviti fizikal (senaman).**

- Ianya bukan untuk menguji pengetahuan anda. Oleh itu, **tiada jawapan yang betul atau salah.**

- Sila pilih **satu jawapan** yang paling **sesuai untuk menggambarkan tahap persetujuan anda** terhadap kenyataan tersebut dengan **membulatkan jawapan** anda.

.

Part III. Perception of cardiovascular diseases (e.g. heart disease/stroke) and CVD health checks

- *The following questionnaire enquires about your perception about various components relating to cardiovascular diseases (CVD) and CVD health check..*

  -Cardiovascular diseases (CVD) in these questions refer to heart attack and stroke.

  - CVD *Health check in the following questions refer to health check for CVD prevention which medical examinations whose purpose is to screen for cardiovascular disease risk factors such as high blood pressure, diabetes, high cholesterol, obesity, smoking and lack of physical activity (exercise).*

- *There is no right or wrong answer.*

- *For each statement below, please circle the scale that best describes your level of agreement or disagreement.*

Skala	Sangat tidak setuju: **STS (1)**	Tidak setuju: **TS (2)**	Tidak pasti : **TP (3)**	Setuju: **S (4)**	Sangat setuju: **SS (5)**

Scale:	*Strongly disagree:* ***SD (1)***	*Disagree:* ***D (2)***	*Not sure :* *N (3)*	*Agree:* *A (4)*	*Strongly agree:* *SA (5)*

Bil	Soalan	Skala Maklumbalas				
		**STS** *SD*	**TS** *D*	**TP** *N*	**S** *A*	**SS** *SA*
A1	Saya percaya penyakit CVD (contohnya penyakit serangan jantung dan strok) dapat dicegah. *I believe CVD (for example heart disease, stroke, etc.) can be prevented.*	1	2	3	4	5
A2	Saya percaya rawatan awal faktor risiko penyakit CVD (contohnya darah tinggi, kolesterol tinggi, kencing manis dan lain-lain) dapat mencegah penyakit CVD. *I believe early treatment of CVD risk factors (for example high blood pressure, high cholesterol level, diabetes mellitus) can prevent the CVD.*	1	2	3	4	5
A3	Saya percaya penyakit CVD dapat dirawat. *I believe CVD is treatable.*	1	2	3	4	5
A4	Jika penyakit CVD dapat dikesan awal, rawatannya adalah lebih senang. *If CVD can be detected early, the treatment will be easier.*	1	2	3	4	5
B1	Saya mempunyai risiko untuk mendapat penyakit CVD. *I am at risk of CVD.*	1	2	3	4	5
B2	Umur saya sekarang menjadikan saya berisiko untuk mendapat penyakit CVD. *My current age puts me at risk of CVD.*	1	2	3	4	5
B3	Gaya hidup saya menjadikan saya berisiko untuk mendapat penyakit CVD. *My lifestyle puts me at risk of CVD.*	1	2	3	4	5
B4	Masalah kesihatan ahli keluarga saya menjadikan saya berisiko untuk mendapat penyakit CVD. *Medical problems in my family members put me at risk of CVD.*	1	2	3	4	5
B5	Keadaan kesihatan saya sekarang menjadikan saya berisiko untuk penyakit CVD. *My current health condition put me at risk of CVD.*	1	2	3	4	5

Skala	Sangat tidak setuju: **STS (1)**	Tidak setuju: **TS (2)**	Tidak pasti : **TP (3)**	Setuju: **S (4)**	Sangat setuju: **SS (5)**

Scale:	*Strongly disagree:* ***SD (1)***	*Disagree:* ***D (2)***	*Neutral :* ***N (3)***	*Agree:* ***A (4)***	*Strongly agree:* ***SA (5)***

**Bil**	**Soalan**	**Skala Maklumbalas**				
		**STS** *SD*	**TS** *D*	**TP** *N*	**S** *A*	**SS** *SA*
Untuk mencegah penyakit kardiovaskular, *For CVD prevention,*						
C2	saya lebih suka mengamalkan gaya hidup sihat berbanding menjalani pemeriksaan kesihatan CVD. *I prefer to adopt a healthy lifestyle than undergoing CVD health check.*	1	2	3	4	5
C3	saya lebih yakin mengamalkan gaya hidup sihat berbanding menggunakan rawatan perubatan. *I am more confident with practising healthy lifestyle than using medical treatment.*	1	2	3	4	5

Skala	Sangat tidak setuju: **STS (1)**	Tidak setuju: **TS (2)**	Tidak pasti : **TP (3)**	Setuju: **S (4)**	Sangat setuju: **SS (5)**

Scale:	*Strongly disagree:* ***SD (1)***	*Disagree:* ***D (2)***	*Neutral :* *N (3)*	*Agree:* *A (4)*	*Strongly agree:* *SA (5)*

Bil	Soalan	Skala Maklumbalas				
		**STS** *SD*	**TS** *D*	**TP** *N*	**S** *A*	**SS** *SA*
DB1	Saya rasa menjalani pemeriksaan kesihatan CVD dapat memberi jaminan kesihatan saya.<br>*I feel undergoing CVD health check will give assurance for my health.*	1	2	3	4	5
DB2	Kita tidak akan tahu tahap kesihatan CVD diri jika tidak menjalani pemeriksaan kesihatan CVD.<br>*We will not know our CVD health status if we do not undergo CVD health checks.*	1	2	3	4	5
DB3	Pemeriksaan kesihatan CVD akan memberi petunjuk untuk pencegahan penyakit CVD.<br>*CVD health check can act as an indicator for CVD prevention.*	1	2	3	4	5
DB4	Pemeriksaan kesihatan CVD membolehkan kita mengesan awal faktor risiko penyakit jantung/strok (contoh darah tinggi, kolesterol tinggi, kencing manis dan lain-lain).<br>*CVD health check enables us to detect risk factors of heart disease/stroke early.*	1	2	3	4	5

Bil	Soalan	Skala Maklumbalas				
		**STS** *SD*	**TS** *D*	**TP** *N*	**S** *A*	**SS** *SA*
Pemeriksaan CVD<br>*CVD health check*						
DD1	adalah pembaziran masa.<br>*is a waste of time.*	1	2	3	4	5
DD2	adalah pembaziran wang.<br>*is a waste of money.*	1	2	3	4	5
DD3	melibatkan prosedur yang menyusahkan. (contohnya: keperluaan berpuasa sebelum pemeriksaan darah).<br>*involves a troublesome procedure. (e.g. the need to fast before blood test.)*	1	2	3	4	5
DD4	yang mendapati keputusan tidak normal akan menimbulkan masalah. (contohnya menjejaskan peluang untuk membeli insurans atau mendapatkan kerja).<br>*which found abnormal health check results will give rise to problems. (e.g. affect the chance to purchase insurance or get a job).*	1	2	3	4	5

Skala	Sangat tidak setuju: **STS (1)**	Tidak setuju: **TS (2)**	Tidak pasti : **TP (3)**	Setuju: **S (4)**	Sangat setuju: **SS (5)**

Scale:	*Strongly disagree:* ***SD (1)***	*Disagree:* ***D (2)***	*Neutral :* *N (3)*	*Agree:* *A (4)*	*Strongly agree:* *SA (5)*

Bil	Soalan	Skala Maklumbalas				
		**STS** *SD*	**TS** *D*	**TP** *N*	**S** *A*	**SS** *SA*
RF R1	Saya bersedia untuk menghadapi keputusan pemeriksaan kesihatan CVD.<br>*I am ready to face the results of the CVD health check.*	1	2	3	4	5
RF R2	Saya ingin mendapat tahu tahap kesihatan CVD saya.<br>*I want to know my CVD health status.*	1	2	3	4	5
RF R3	Saya tidak mahu berfikir dan tidak ingin mengambil tahu langsung mengenai penyakit CVD.<br>*I don't want to think and know about CVD diseases at all.*	1	2	3	4	5

Bil	Soalan	Skala Maklumbalas				
		**STS** *SD*	**TS** *D*	**TP** *N*	**S** *A*	**SS** *SA*
Jika keputusan pemeriksaan kesihatan CVD tidak normal,<br>*If the CVD health check results are abnormal,*						
RH O1	saya bersedia untuk mengambil rawatan ubat.<br>*I am ready to take medication.*	1	2	3	4	5
RH O2	saya bersedia untuk mengubah gaya hidup saya.<br>*I am ready to adjust my lifestyle.*	1	2	3	4	5
RH O3	saya bersedia untuk menanggung kos rawatan berikutnya.<br>*I am ready to bear the cost of subsequent treatment.*	1	2	3	4	5
RH O4	Saya tidak bersedia untuk melakukan apa-apa.<br>*I am not ready to do anything.*					

Skala	Sangat tidak setuju: **STS (1)**	Tidak setuju: **TS (2)**	Tidak pasti : **TP (3)**	Setuju: **S (4)**	Sangat setuju: **SS (5)**

Scale:	*Strongly disagree:* ***SD (1)***	*Disagree:* ***D (2)***	*Neutral :* ***N (3)***	*Agree:* ***A (4)***	*Strongly agree:* ***SA (5)***

**Bil**	**Soalan**	**Skala Maklumbalas**				
		**STS** *SD*	**TS** *D*	**TP** *N*	**S** *A*	**SS** *SA*
F1	Saya akan berusaha untuk meluangkan masa untuk menjalani pemeriksaan kesihatan CVD.<br>*I will make the effort to allocate time to go for a CVD health check.*	1	2	3	4	5
F2	Bagi saya, kos pemeriksaan kesihatan CVD adalah membebankan.<br>*The cost of doing a CVD health checks is a burden for me.*	1	2	3	4	5
F3	Tempat pemeriksaan kesihatan CVD adalah jauh dari rumah/tempat kerja saya.<br>*The place for CVD health checks is far from my house/workplace.*	1	2	3	4	5
F4	Saya ada masalah pengangkutan untuk pergi menjalani pemeriksaan kesihatan CVD.<br>*I have a problem with transportation to go for CVD health check .*	1	2	3	4	5

**Bil**	**Soalan**	**Skala Maklumbalas**				
		**STS** *SD*	**TS** *D*	**TP** *N*	**S** *A*	**SS** *SA*
Saya akan menjalanin pemeriksaan kesihatan CVD<br>*I will perform the CVD health check*						
G1	jika dinasihatkan oleh doktor.<br>*if recommended to do so by doctors.*	1	2	3	4	5
G2	jika dinasihatkan oleh ahli keluarga saya.<br>*if my family member advises me to do so.*	1	2	3	4	5
G3	jika dinasihatkan oleh kawan saya.<br>*if my friend advises me to do so.*	1	2	3	4	5
G4	jika majikan memerlukan saya berbuat demikian.<br>*if my employer requires me to do so.*	1	2	3	4	5
G5	kerana orang-orang sekeliling saya telah berbuat demikian.<br>*as people around me have already done so.*	1	2	3	4	5

Bahagian IV. Keinginan untuk menjalani pemeriksaan kesihatan CVD
Kenyataan berikut berkenaan dengan keinginan/niat anda untuk menjalani pemeriksaan kesihatan untuk pencegahan penyakit kardiovaskular.
Sila pilih **satu jawapan** yang paling sesuai untuk menggambarkan tahap persetujuan anda terhadap kenyataan tersebut dengan **membulatkan jawapan** anda.

Part IV. The intention to undergo CVD health checks.
The following statement is about your intention to participate in the health check for CVD prevention.
For each statement below, please circle the scale that best describes your level of intention.

Skala<br>*Scale*	Sangat tidak mungin:<br>**STM (1)**	Tidak mungkin:<br>**TM (2)**	Tidak pasti :<br>**TP (3)**	Mungkin :<br>**M (4)**	Sangat mungkin:<br>**SM (5)**

Scale	Very unlikely:<br>**VUL (1)**	Unlikely:<br>**UL (2)**	Not sure :<br>**NS (3)**	Likely:<br>**L (4)**	Very likely:<br>**VL (5)**

**Bil**<br>**no**	**Soalan**<br>**Item**	**Skala Maklumbalas** **Response Scale**				
		**STM**<br>***VUL***	**TM**<br>***UL***	**TP**<br>***NS***	**M**<br>***L***	**SM**<br>***VL***
1	Apa kemungkinan anda untuk pergi menjalani pemeriksaan kesihatan CVD dalam masa berikut:<br>*How likely are you going to undergo CVD health checks within the following time period:*					
	A) Dalam 3 bulan yang akan datang?<br>Within the next 3 months?	1	2	3	4	5
	B) Dalam 6 bulan yang akan datang?<br>Within the next 6 months?	1	2	3	4	5
	C) Dalam tempoh 1 tahun yang akan datang?<br>Within the next 1 year?	1	2	3	4	5

Terima kasih di atas kerjasama anda.
Thank you for your participation.
