# Supplementary material for: What are the determinants for individuals to undergo cardiovascular disease health checks? A cross sectional survey
Source: PLoS One. 2018 Aug 9;13(8):e0201931. doi: 10.1371/journal.pone.0201931 (PMC6085058; doi:10.1371/journal.pone.0201931)
Supplement: S1 Table — (PDF) [file pone.0201931.s002.pdf]

S1 Table

Table 1: Classification of the degree of likeliness to undergo health check

Category	The highest score regardless of timeline	Categories used in ordinal regression
Very unlikely	1	Very unlikely, unlikely or not sure
Unlikely	2	
Not sure	3	
Likely	4	Likely
Very likely	5	Very likely

Table 2: Classification of likely timeline to undergo health checks

Category	Definition
Not sure or not likely to attend	Score of 1, 2 or 3 for all the time period
Likely to attend within one year	Score of 4 or 5 within 1 year
Likely to attend within 6 months	Score of 4 or 5 within 6 months
Likely to attend within 3 months	Score of 4 or 5 within 3 months
