# Supplementary material for: What are the determinants for individuals to undergo cardiovascular disease health checks? A cross sectional survey
Source: PLoS One. 2018 Aug 9;13(8):e0201931. doi: 10.1371/journal.pone.0201931 (PMC6085058; doi:10.1371/journal.pone.0201931)
Supplement: S2 Table — (PDF) [file pone.0201931.s003.pdf]

S2 Table

Table 1 Summary results of pseudo-R^2 and test of parallel lines for four models without control of sociodemography

Models	Outcome variable	n	Nagelkerke (pseudo-R^2)	Test of parallel lines	
				-2Log-Likelihood χ^2	p
1	Degree of likeliness to undergo CVD health checks	413	0.227	7.679	0.567
2	Likely timeline of the public to undergo CVD health checks	413	0.163	26.855	0.082
3	Degree of likeliness to undergo CVD health checks	397	0.248	9.304	0.410
4	Likely timeline of the public to undergo CVD health checks	397	0.178	27.827	0.065

Table 2 Summary results of pseudo-R2 , and test of parallel lines for four models with control of sociodemography

Models	Outcome variable	n	Nagelkerke ($pseudo-R^2$)	Test of parallel lines	
				-2Log-Likelihood χ^2	p
1	Degree of likeliness to undergo CVD health checks	412	0.429	26.906	0.174
2	Likely timeline of the public to undergo CVD health checks	412	0.279	54.278	0.097
3	Degree of likeliness to undergo CVD health checks	396	0.453	26.173	0.200
4	Likely timeline of the public to undergo CVD health checks	396	0.301	64.866	**0.013**
