# Supplementary material for: What are the determinants for individuals to undergo cardiovascular disease health checks? A cross sectional survey
Source: PLoS One. 2018 Aug 9;13(8):e0201931. doi: 10.1371/journal.pone.0201931 (PMC6085058; doi:10.1371/journal.pone.0201931)
Supplement: S3 Table — (PDF) [file pone.0201931.s004.pdf]

S3 Table

Table 1: Correlation matrix for determinant variables in the model

	1	2	3	4	5	6	7	8	9
1	1.000	0.105	0.601	0.431	-0.274	0.394	0.307	-0.311	0.261
2		1.000	0.052	0.200	0.025	0.134	0.044	-0.075	0.163
3			1.000	0.224	0.080	0.074	0.072	0.031	0.168
4				1.000	-0.338	0.496	0.365	-0.314	0.409
5					1.000	-0.484	-0.364	0.364	-0.256
6						1.000	0.483	-0.417	0.377
7							1.000	-0.485	0.349
8								1.000	-0.297
9									1.000

1: Believe that the disease course can be changed for better outcomes
2: Perceived self at risk of CVD
3: Preferred method for CVD prevention
4: Perceived benefits of health checks
5: Perceived drawbacks of health checks
6: Readiness to know the result of health checks
7: Readiness to handle the outcomes following health checks
8: External barriers
9: Influence by significant others
